# New Challenges and Perspectives in Neurology and Autonomic Disorders: A Leap Forward

**DOI:** 10.3390/jpm14101063

**Published:** 2024-10-16

**Authors:** Svetlana Blitshteyn, Ilene Ruhoy

**Affiliations:** 1 Department of Neurology, School of Medicine and Biomedical Sciences, University of Buffalo Jacobs, Buffalo, NY 14203, USA; 2 Dysautonomia Clinic, Williamsville, NY 14221, USA; 3 Department of Neurology, Mount Sinai South Nassau, Oceanside, NY 11572, USA

“Nothing in life is to be feared, it is only to be understood. Now is the time to understand more so that we may fear less.”

—Marie Curie

The autonomic nervous system, which consists of the sympathetic, parasympathetic, and enteric divisions, is an integral part of the central and peripheral nervous systems and controls homeostasis, blood flow, and responses to internal and external stimuli. Disorders of the autonomic nervous system—both common, such as postural orthostatic tachycardia syndrome (POTS), neurocardiogenic syncope, and orthostatic hypotension (OH); and rare, such as multiple system atrophy, amyloid neuropathy, and familial dysautonomia—have been an evolving area of research in basic and translational science as well as in clinical practice. More recently, the COVID-19 pandemic further underscored the need to elucidate the neurologic and autonomic mechanisms of post-infectious syndromes. To this end, the new frontier in neurology and autonomic disorders, as well as the mechanistic interplay between a wide range of neurologic conditions and autonomic dysfunctions, present an exciting opportunity for groundbreaking discoveries.

In this Special Issue, we aimed to collect original research, reviews, hypothesis, perspectives, and opinions on autonomic disorders and how it affects various medical subspecialties, including neurology, cardiology, infection-associated chronic illnesses, headache medicine, psychiatry, and others. We were particularly interested in studies and reviews of the potential biomarkers and identification of effective diagnostic and therapeutic approaches in patients with complex neurologic and autonomic disorders and how they advance our understanding of these disorders, in addition to helping us expand our diagnostic and therapeutic capabilities in clinical practice.

It has been known from clinical observations and some experimental studies that the autonomic nervous system extends beyond the regulation of the target organs by the parasympathetic, sympathetic, and enteric nervous systems and that it closely communicates with the immunologic system and inflammatory pathways. David Goldstein, MD, Ph.D., Chief of Autonomic Section at the National Institutes of Health, provides a perspective on the “extended” autonomic system (EAS) and the “homeostat” theory as applied to the pathophysiology and potential treatments of dysautonomia (contribution 1). He emphasizes that the ANS may include neuroendocrine, immune/inflammatory, and central components and that comparators in the form of thermostat, glucostat, carbistat, and barostat exist that regulate different variables, such as core temperature, blood glucose, blood gases, and delivery of blood to the brain. He presents the homeostat theory and how it applies to EAS with specific examples of pediatric, adolescent/adult, and geriatric forms of dysautonomia and argues that computer modeling has the potential to lead to individualized treatments and outcomes (contribution 1).

The neuropsychiatric manifestations of systemic disease are an under-represented area of research in neurology that deserve increased research interest, education time, and neurology training. Weinstock et al. reported a case series of eight patients with mast cell activation syndrome (MCAS)—a multisystemic immunologic disorder with an estimated prevalence of 17%—who experienced significant neuropsychiatric disorders that were refractory to standard therapies. Five patients had depression, five had generalized anxiety disorder, and four had a panic disorder (contribution 2). All eight patients were subsequently diagnosed with MCAS; six out of eight patients had comorbid autonomic disorders with the most common being POTS, and four had hypermobile Ehlers–Danlos syndrome (h-EDS). All patients experienced significant improvement in their neuropsychiatric and multisystemic symptoms after mast-cell-directed therapy was implemented, which included antihistamines, mast-cell-stabilizing agents and a low-histamine diet (contribution 2). This case series illustrates the systemic nature of common neurologic and psychiatric disorders, which need to be identified and treated.

Migraine is one of the most comorbidities of POTS, with both disorders being heavily influenced by sex hormones. Godley III et al. review how sex hormones affect migraine with the help of interdisciplinary research scientists that focused on examining estrogen and oxytocin while noting that progesterone, testosterone, and vasopressin were less well-studied (contribution 3). They conclude that progress in research on the effects of hormones on the nervous system has been slow and that substantial gaps exist in our understanding of the complex roles sex hormones play in migraine (contribution 3). Increased funding, interdisciplinary research efforts, and exploring therapeutic agents, such as oxytocin delivered via nasal spray, could advance the science and therapeutic implications of sex hormones in women with migraine at various stages of life.

Long COVID-19 highlighted a wide gap in our understanding and clinical approach to patients with post-acute infection syndromes, which commonly include POTS and myalgic encephalomyelitis/chronic fatigue syndrome (ME/CFS). However, with the renewed interest and investment in the field of infection-associated chronic illnesses, we have made significant progress in our understanding of the complex pathophysiology of post-acute sequelae of SARS-CoV-2, which includes autonomic dysfunction, immune dysregulation, endothelial disturbance, vascular and neuropathic changes, microbiome alteration, microglial activation, blood–brain barrier disruption and other pathologic manifestations [1]. Importantly, Davenport et al. emphasize that ong COVID-19 is not a functional neurologic disorder (FND) (contribution 4). FND is previously known as conversion disorder and hysteria—a condition rooted in psychosocial etiology and distorted sense of agency and emotional processing, which is commonly treated with FND-targed psychotherapy and physical therapy. The authors assert that the vast majority of patients with long COVID-19 do not have FND, but do have dysautonomia and ME/CFS, which should not be mislabeled with FND as pathophysiology, diagnostic tests, physical exam, and treatment approaches are significantly different between these disorders (contribution 4).

Adding to the expanding science on long COVID-19, Tabacof et al. reviewed echocardiograms of over 200 patients with post-COVID-19 dysautonomia and queried if these symptoms may be cardiogenic (contribution 5). They found that most patients did not show evidence of cardiac abnormalities on echocardiography. Interestingly, they found that patients with post-COVID-19 dysautonomia had lower stroke volume than in an unclassified subgroup and that stroke volume and left-ventricular end-diastolic volume were smaller in those reporting decreased physical activity after COVID-19 (contribution 5). Similar findings were identified in patients with POTS before the COVID-19 pandemic [2,3].

Hypercoagulable state has been found in many patients with long COVID-19 and some patients with POTS. Kell and Pretorius et al. argue that fibrinaloid microclots may be important in the pathophysiology of POTS through their ability to block the flow of blood through microcapillaries and thus cause tissue hypoperfusion (contribution 6). Amyloids are known to be membrane disruptors and may affect the autonomic nerve fibers. Previously, they showed the presence of microclots in patients with long COVID-19 [4]—a finding that was recently confirmed by another study demonstrating that fibrin drives the thromboinflammation and neuropathology after COVID-19 infection [5]. It remains to be determined whether the same mechanisms are involved in POTS, especially in the context of post-acute infection syndromes.

Current research highlight autoimmunity as an important pathophysiologic mechanism of POTS and its numerous comorbidities, including gastrointestinal disorders. Nakane et al. examined patients diagnosed with functional gastrointestinal disorders and found that among 11 patients with irritable bowel syndrome and functional dyspepsia, 4 had anti-ganglionic nicotinic acetylcholine receptor antibodies measured via luciferase immunoprecipitation system assay, with 3 also having dry eyes and dry mouth, while there were no such symptoms in antibody-negative group (contribution 7). Further studies are needed to determine the prevalence of these and other antibodies in patients with functional gastrointestinal disorders.

Continuing the important topic of autoimmunity in autonomic disorders, Pena et al. offered a comprehensive literature review on a variety of autoantibodies and immunomodulatory therapies that have been described in patients with POTS and OH (contribution 8). They highlight the existing studies and case series that demonstrate the presence of antinuclear, anti-phospholipid, alpha and beta adrenergic, cholinergic, and angiotensin II type I autoantibodies associated with POTS and OH. Importantly, case reports and series suggest that immunotherapy with intravenous and subcutaneous immunoglobulin as well as plasmapheresis can be beneficial in patients with severe POTS refractory to standard therapies (contribution 8). Large clinical trials, including the NIH RECOVER-AUTONOMIC trial assessing the benefits of IVIG, are currently in progress to determine the efficacy of these therapies in patients with post-COVID POTS and autonomic dysfunction [6].

Diagnostic testing is an integral part of the clinical evaluation and diagnostic criteria of autonomic disorders. Jason et al. conducted a study of 193 patients with ME/CFS using a tilt table test whereas 32.5% of patients in this cohort tested positive for POTS or OH (contribution 9). The participants with either of these two common autonomic disorders were found to have more problems with sleep and post-exertional malaise as well as greater physical and health function limitations. These findings highlight the need for further understanding of the etiology of symptoms that have been ascribed to POTS or OH, how the symptoms may or may not interfere in the interpretation of the TTT, and what other tests (that are more sensitive and specific for autonomic dysfunction) should be developed for patients with clear autonomic dysfunction, but a negative tilt table test.

Perfecting simple and non-invasive means of testing for objective data and improved diagnosis and thus treatment options for patients with Parkinson’s disease, Fernando et al. found that having patients with Parkinson’s disease take their blood pressure at home twice daily in lying and standing positions over 5 days greatly improved identification of blood pressure disturbance, including OH, in comparison to a single in-office measurement (contribution 10). This may help with early recognition of OH and other blood pressure disturbances, as well as implementation of interventions to mitigate dysautonomia, in patients with Parkinson’s disease—one of the most common movement disorders seen in neurology clinics.

This Special Issue is only a tiny particle in the universe of the unknown about the brain and the autonomic nervous system. Nevertheless, we hope it can serve as an important source of information that generates excitement, curiosity, and further research in neurology, neuroscience, and interdisciplinary specialties. It has been an honor and a privilege for us to serve as the Guest Editors of this Special Issue, and to read, edit, and learn from the innovative, cutting-edge contributions, written by some of the best researchers, clinicians, and world-renowned experts in the field. As neurology and autonomic disorders advance and as we continue to face the COVID-19 pandemic with increased prevalence of post-COVID-19 complications, investigating the pathophysiology, diagnostic tests, and therapeutic options for complex neurologic and autonomic disorders becomes our top priority. It is a leap forward that we must take in our quest for scientific inquiry and advancement in medicine, science, patient care, and public health.
